# Integration of Machine Learning and Structural Analysis for Predicting Peptide Antibiofilm Effects: Advancements in Drug Discovery for Biofilm-Related Infections

**DOI:** 10.5812/ijpr-138704

**Published:** 2023-09-30

**Authors:** Fatemeh Ebrahimi Tarki, Mahboobeh Zarrabi, Ahya Abdiali, Mahkame Sharbatdar

**Affiliations:** 1 Department of Biotechnology, Faculty of Biological Sciences, Alzahra University, Tehran, Iran; 2 Department of Microbiology, Faculty of Biological Sciences, Alzahra University, Tehran, Iran; 3 Department of Mechanical Engineering, Khajeh Nasir Toosi University of Technology, Tehran, Iran

**Keywords:** Antibiotics Resistance, Biofilm Inhibition, Peptides, Supervised Machine Learning

## Abstract

**Background:**

The rise of antibiotic resistance has become a major concern, signaling the end of the golden age of antibiotics. Bacterial biofilms, which exhibit high resistance to antibiotics, significantly contribute to the emergence of antibiotic resistance. Therefore, there is an urgent need to discover new therapeutic agents with specific characteristics to effectively combat biofilm-related infections. Studies have shown the promising potential of peptides as antimicrobial agents.

**Objectives:**

This study aimed to establish a cost-effective and streamlined computational method for predicting the antibiofilm effects of peptides. This method can assist in addressing the intricate challenge of designing peptides with strong antibiofilm properties, a task that can be both challenging and costly.

**Methods:**

A positive library, consisting of peptide sequences with antibiofilm activity exceeding 50%, was assembled, along with a negative library containing quorum-sensing peptides. For each peptide sequence, feature vectors were calculated, while considering the primary structure, the order of amino acids, their physicochemical properties, and their distributions. Multiple supervised learning algorithms were used to classify peptides with significant antibiofilm effects for subsequent experimental evaluations.

**Results:**

The computational approach exhibited high accuracy in predicting the antibiofilm effects of peptides, with accuracy, precision, Matthew's correlation coefficient (MCC), and F1 score of 99%, 99%, 0.97, and 0.99, respectively. The performance level of this computational approach was comparable to that of previous methods. This study introduced a novel approach by combining the feature space with high antibiofilm activity.

**Conclusions:**

In this study, a reliable and cost-effective method was developed for predicting the antibiofilm effects of peptides using a computational approach. This approach allows for the identification of peptide sequences with substantial antibiofilm activities for further experimental investigations. Accessible source codes and raw data of this study can be found online (hiABF), providing easy access and enabling future updates.

## 1. Background

In recent decades, there have been significant changes in the nature of infectious diseases. According to a study by Batoni et al., infections that were prevalent until the mid-twentieth century were characterized by sudden onset, responsiveness to antibiotic treatment, well-defined pathogenic mechanisms, and the ability to isolate pathogenic agents from infected tissues. However, today, infections are characterized by chronic progression, resistance to antimicrobials, unknown pathogenic mechanisms, and the potential involvement of normal flora microorganisms (1).

**Figure 1.**
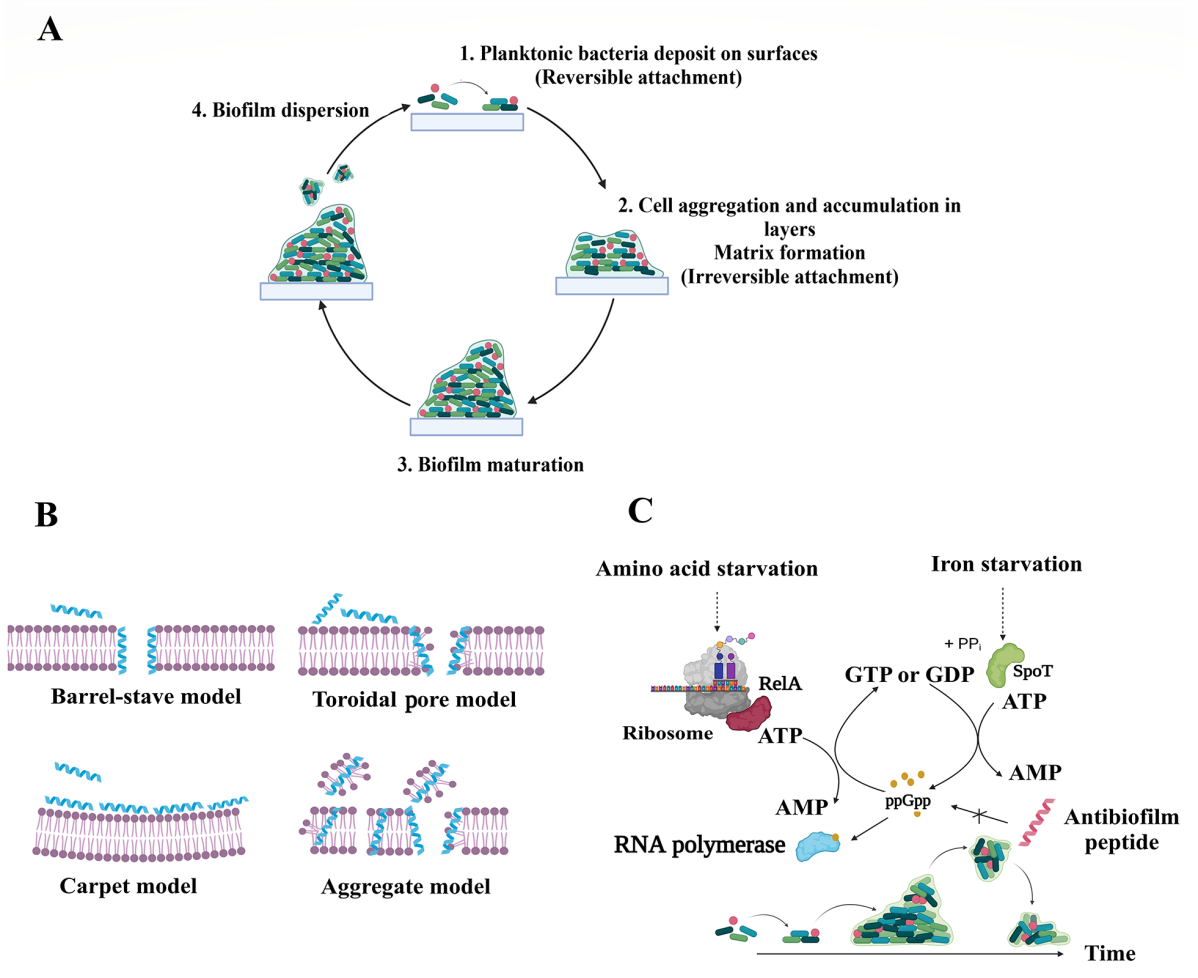
Biofilm formation steps (A); two main mechanisms of action of antibiofilm (ABF) agents include membrane-lytic peptides (B); and interference with ppGpp signaling process (C)

Bacterial biofilms play an important role in the emergence of antibiotic resistance, as biofilm-producing bacteria exhibit higher resistance to almost all available antibiotics compared to planktonic bacteria (2). The ability of biofilms to form on various surfaces, including tissues and medical devices, poses a significant challenge in healthcare systems (2). Biofilm formation involves three stages, including adhesion through weak and reversible long-range interactions, maturation through the production of extracellular polymeric substances (EPS) that maintain structural integrity, and dispersal through cell detachment and colonization of nearby sites (3). Generally, biofilm is a complex and dynamic system, distinguished by its formation process, the presence of EPS, and a unique distribution of molecules, such as nutrients and oxygen, resulting in a heterogeneous structure in terms of temporal and spatial organization (1). Figure 1A demonstrates a schematic representation of biofilm formation.

It is known that in biofilms, bacteria develop resistance mechanisms through reduced diffusion and sequestration of antimicrobials by EPS, slow growth rates, and the presence of dormant cells, known as persister cells (4). Given the importance of these specific features in the development of antibiofilm (ABF) agents, utilizing nature-based therapeutics, such as peptides, is a practical approach to addressing the rise of antibiotic resistance (5). Ideal ABF agents should possess characteristics, such as rapid killing ability, effectiveness in various environments, the ability to penetrate EPS, and synergistic interactions with other conventional or non-conventional antimicrobial agents (1).

Antimicrobial peptides (AMPs), also known as host defense peptides (HDPs), are found in all forms of life, including bacteria, plants, vertebrates, and invertebrates (6). They demonstrate a broad range of antibacterial (ABP), antifungal, antiviral, anticancer, antiparasitic, and ABF effects and are classified based on their activity (7). It has been shown that ABF agents have some characteristics of ideal ABF agents. In this regard, Raheem and Straus conducted an all-inclusive review of ABF mechanisms of action (8). Antibiofilm agents primarily target bacterial membranes and are attracted to their negatively charged surfaces through electrostatic interactions. Meanwhile, other mechanisms of action have been reported for ABFs, such as the inhibition of the stringent response signaling nucleotide, ppGpp (9). The stringent response plays a crucial role in the development and maintenance of biofilms in both Gram-positive and Gram-negative bacteria (8). Figure 1B and C illustrate these two main mechanisms of action of ABF agents.

Factors, such as high efficiency, simplicity of synthesis, short market entry time, and well-understood mechanisms of action, have made ABF biomolecules attractive candidates for drug development. However, it is important to address challenges, such as enhancing specificity to avoid side effects when developing ABF-based drugs (10). The optimal characteristics of peptides for targeting planktonic bacteria may not be the same as those required for combating bacteria in a biofilm (1). Therefore, a thorough understanding of the key characteristics of peptide sequences that are essential for their ABF effects can significantly enhance their performance.

In the field of drug development, there has been a growing interest in the utilization of artificial intelligence algorithms, particularly machine learning and deep learning approaches (11). These approaches offer several advantages over experimental methods, including cost-effectiveness and time-saving capabilities, which can help reduce certain burdens (12). Given the need to automate repetitive data processing and analysis tasks in drug discovery research, the integration of machine learning techniques capable of identifying patterns in large datasets has become essential.

Machine learning techniques have been applied to predict various peptide activities, including anticancer, antimicrobial, hemolytic, and ABF activities (13-16). In this regard, Sharma et al. used a positive library consisting of 80 ABF peptides, as well as a negative library, consisting of quorum-sensing peptides (QSPs), which play an auto-inducing role in biofilm development in Gram-positive bacteria without an ABF activity. They trained support vector machine (SVM) and Weka-based systems, achieving a maximum accuracy of 95.24% and a Matthew's correlation coefficient (MCC) of 0.91 on the training dataset (17). Additionally, Gupta et al. selected 178 ABF peptides as the positive library and randomly generated peptide sequences from the Swiss-Prot database as the negative library. Their SVM-based model exhibited an accuracy of 97.18% and an MCC of 0.84 on the validation dataset (18). Moreover, Fallah Atanaki et al. developed SVM and random forest-based models, with positive and negative libraries consisting of 178 and 88 ABF peptides and QSPs, respectively. Their SVM-based model exhibited an accuracy of 95% and an MCC of 0.89 (16).

Overall, the abovementioned studies all play a significant role in the recognition and prediction of the ABF effects of peptides. Nevertheless, analyzing the ABF activity of peptides is an important goal that should have been taken into account in these studies. Therefore, the focus of the present study was to gain a comprehensive understanding of the structure and properties of highly active antibiofilm (hiABF) peptides. For this purpose, a machine learning-based method with improved performance was developed. A positive library was assembled, consisting of experimentally validated peptide sequences with ABF activity exceeding 50%. Also, a negative library was constructed using QSPs. Features based on physicochemical properties, amino acid composition, sequence order, and the distribution of physicochemical properties were calculated to create a feature space.

## 2. Objectives

In the present study, compositional and characteristic analyses focused on three categories, and comparisons were made between hiABFs and QSPs, between hiABFs and antibacterial peptide sequences (ABPs), and between hiABFs that act on preformed biofilms and those that inhibit biofilm formation. Additionally, the positional preference of amino acids at the N and C terminals of hiABFs was analyzed, although this privileged information was not used for training the models. To the best of our knowledge, this combination of features was used for the first time in the present study to map peptide sequences to numeric feature vectors, with a focus on peptides exhibiting ABF activity more than 50%. In this study, a computational approach was developed to accurately predict the ABF effects of peptides with a high level of accuracy. This approach can help address the challenges and expenses of designing peptides with significant ABF effects, and the findings can lead to the development of new therapeutic agents to combat antibiotic resistance.

## 3. Methods

### 3.1. Dataset Collection

The BaAMPs database, which is publicly accessible, was used to prepare peptides with ABF effects for the positive library (19). Peptide sequences with ABF activity exceeding 50% were selected by browsing the database based on activity. It is worth noting that the BaAMP database was last updated in 2014. To broaden the dataset, an extensive analysis of 394 results from the Web of Science was conducted in the period of 2014 - 2022, using the methodology and keywords outlined by Di Luca et al. (19). The analysis resulted in the identification of peptide sequences with an ABF activity of 50% or higher.

**Figure 2.**
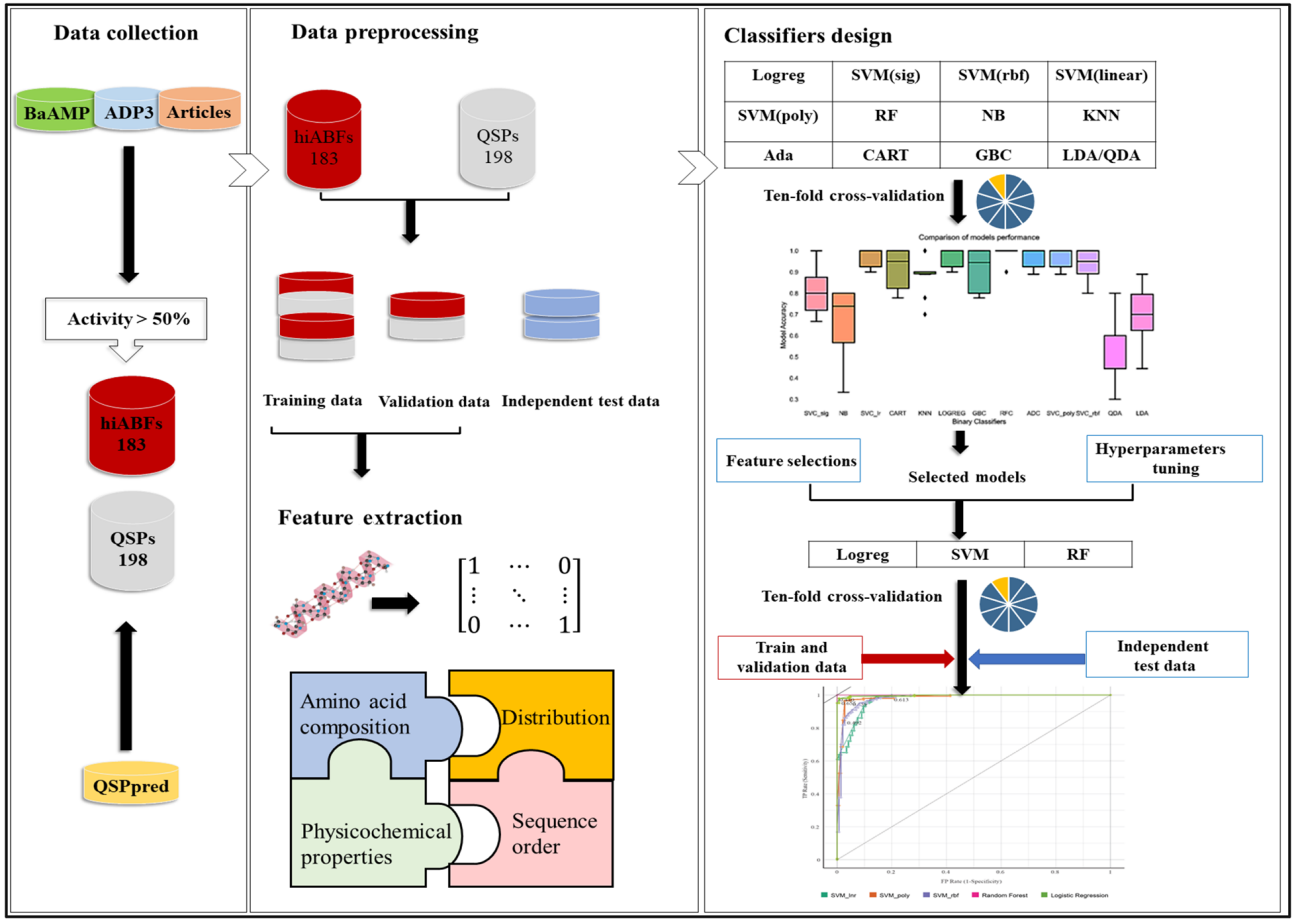
A flowchart of library preparation and classifier design stages

Moreover, the APD3 database was evaluated with the same approaches (20). Sequences with high ABF activity, which were not separated in the initial database, as well as relevant articles, were added to the positive library. Finally, the positive library was prepared with 183 peptide sequences with an ABF activity exceeding 50%. To construct the negative library, peptide sequences that acted as inducers in the quorum-sensing process of Gram-positive bacteria were used. The method presented by Wei et al. was employed to prepare a negative library, containing 198 QSPs (21). Duplicated sequences and sequences containing non-standard amino acid residues, modifications, or indefinite residues were excluded from both positive and negative libraries. A flowchart illustrating the steps of library preparation and classifier design is presented in Figure 2. 

To mitigate bias, the data were randomly divided into three datasets of training, validation, and independent test sets. Overall, 75% of data were used for model training, 48 sequences were used for validation, and 48 sequences were set aside as the independent test set.

### 3.2. Peptide Feature Extraction and Encoding

The conversion of peptide sequences into numerical vectors was performed to construct the feature spaces for supervised learning-based models. In this study, four classes of features were calculated, including amino acid composition, amino acid order, physicochemical properties, and distribution. The features, along with their respective numbers, are presented in Table 1. All the features were obtained using the python-based packages, including iFeatureOmega and modlAMP (22, 23).

**Table 1.** The List of Feature Vectors in Each Category and the Number of Features in Each Class

Feature Class	Feature Name	Number of Calculated Features
**AAC**	AAC and DPC	420
**Distribution**	CTD, CKSAAP, CKSAAGP, and GAAC	1900
**Order**	QSOrder, PAAC, and APAAC	90
**Physicochemical properties**	Globaldesc and PepDESC	56

Abbreviations: AAC, amino acid composition; DPC, dipeptide composition; CTD, composition transition and distribution; QSOrder, quasi-sequence order; PAAC, pseudo amino acid composition; APAAC, amphiphilic pseudo-amino acid composition.

Amino acid composition (AAC) refers to the frequency of each of the 20 natural amino acids in a peptide sequence (24). The formula for calculating AAC is presented in Equation 1:


Equation 1. $$AAC\left(i\right)=\;\frac{aa\left(i\right)}{L}\times 100$$

Where aa(i) is the frequency of each standard amino acid residue, and L represents the peptide length.

Dipeptide composition (DPC) refers to the frequency of dipeptides in a given peptide sequence and is calculated based on Equation 2 (22):


Equation 2. $$D\left(r,s\right)=\frac{N_{rs}}{N-1};\;r,s\;\epsilon \;\{A,C,D,\ldots ,Y\}$$

Where N_rs_ represents the number of times the dipeptide rs appears in the sequence, and N represents the total number of dipeptides. Variables r and s can be any of the 20 standard amino acids.

The composition transition and distribution (CTD) is a feature that indicates the distribution patterns of amino acids with specific structural or physicochemical properties in peptide sequences (25). Attributes, such as hydrophobicity, normalized van der Waals volume, polarity, polarizability, charge, secondary structure, and solvent accessibility, are considered in the calculation of CTD. Generally, amino acids are classified into three groups (1, 2, and 3), based on their properties. For example, peptides can be classified into three groups of coil, helix, and strand, based on their secondary structure (22).

Composition (C) is determined according to Equation 3 which calculates the percentage of each group in the peptide sequence (25). Transition (T) is calculated based on Equation 4 which determines the frequency of transitions from one group to another. Distribution (D) describes the distribution of each attribute in peptide sequences. Five distribution-related descriptors are calculated based on the positional percentage of residues at five key points: The first (0%), 25%, 50%, 75%, and 100% of the sequence (16). In Appendix 1, amino acid attributes and divisions are presented.


Equation 3. $$C=\frac{N_{\left(c\right)}}{N};\;c\;\epsilon \;\left\{1,2,3\right\}$$

Where N is the length of the sequence, and N_(c)_ is the number of c in the encoded sequence.


Equation 4. $$T_{cr}=\frac{N_{cr}+N_{rc}}{N-1};\;cr\;\epsilon \;\left\{\left(1,2\right),\left(1,3\right),\left(2,3\right)\right\}$$

Where N_cr_ and N_rc_ are the numbers of dipeptides encoded as cr and rc, respectively, and N is the length of the sequence.

CKSAAP-CKSAAGP: CKSAAP represents the frequency of dipeptide composition, separated by k amino acid residues (26). CKSAAGP, developed by Chen et al., calculates the frequency of group composition of amino acid pairs, separated by k residues, such as aliphatic-aliphatic or aliphatic-aromatic pairs (22). In this study, the values of k were considered to be 1, 2, and 3.

GAAC: To calculate GAAC, a total of 20 standard amino acids are divided into five classes of aliphatic, aromatic, positive, negative, and uncharged. The frequency of each of these five classes is calculated based on Equation 5 (24):


Equation 5. $$G_{ci}=\frac{N_{ci}}{N};\;ci\;\epsilon \;\left\{c1,c2,c3,c4,c5\right\}$$

Where N_ci_ is the quantity of amino acids in class ci, and N is the length of peptide sequences.

Quasi-sequence order (QSOrder), pseudo amino acid composition (PAAC), and amphiphilic pseudo-amino acid composition (APAAC): To encode peptides comprehensively, it is important to consider both positional and compositional information of amino acids due to their importance in protein and peptide sequence analyses. Two popular methods for encoding amino acid information include QSOrder and PAAC, developed based on research by Chou (27, 28). In addition to composition and order, APAAC also incorporates the hydrophilic and hydrophobic properties of amino acids (29). Appendix 2 depicts a schematic presentation of the order and composition of a peptide sequence with a length of N.

Physicochemical properties: Globaldesc utilizes functions for calculating various physicochemical properties of peptide sequence, including length, charge, charge density, molecular weight, isoelectric point, Boman index (30), instability index (31), aliphatic index (32), aromaticity (33), and hydrophobic ratio (34). Instead of atom-based properties, Zimmerman et al. proposed the Amino-Acid scale (AA-scale), where the side chain of amino acid is assigned a specific value based on its properties. These values are then processed to obtain the final descriptive value for the entire molecule (35). The PepDESC class uses Moreau-Broto correlation functions with variable sliding sequence windows to compute property-based descriptors, such as bulkiness, flexibility, and transmembrane propensity (23). The complete list of global and peptide descriptors is presented in Appendix 3.

### 3.3. Dataset Preprocessing

Before training the models, the datasets were preprocessed to remove variables that were duplicated, had missing values, or had zero variance. All datasets were normalized according to Equation 6 (15):


Equation 6. $$X=\frac{\left(x-x_{min}\right)}{\left(x_{max}-x_{min}\right)}$$

Where x is the value of each calculated feature in a dataset.

### 3.4. Feature Selection

Feature selection is a crucial procedure for building a prediction model by selecting a relevant subset of features while avoiding redundant and irrelevant variables that can negatively affect learning approaches (24). This study applied filter- and wrapper-based methods for creating the feature space. Multicollinearity (MC) was addressed based on a correlation cutoff point of 0.75. Highly correlated features were eliminated, while non-redundant descriptors were retained (36). To select smaller subsets of features, fit them to the model, and remove the weakest features until the final number of features was achieved, recursive feature elimination with cross-validation (RFECV) was employed (15). The SelectKBest method was also utilized to select the best K features with the highest impact on the model’s classification performance (37). Additionally, t-distributed stochastic neighbor embedding (t-SNE), a dimensionality reduction technique, was applied to visualize the selected features using the scikit-learn 0.24.1 library in Python (37, 38).

### 3.5. Machine Leaning Model Architecture

A total of 13 binary classifiers were evaluated, including logistic regression, K-nearest neighbor, decision tree, random forest, gradient boosting classifier, AdaBoost classifier, linear discriminant analysis, quadratic discriminant analysis, naïve Bayes classifier, and SVMs with different kernels, using 10-fold cross-validation on the training and validation datasets. Generally, the K-fold cross-validation method is proper when positive and negative libraries are balanced. In this study, a 10-fold cross-validation approach was employed, considering the size of positive and negative libraries associated with each model (39-47).

Based on the classifier performance, five models yielding superior outcomes were selected for further analysis. Feature selection methods were applied, and a vector space was constructed. To fine-tune the hyperparameters of the model, a grid search with cross-validation (GridSearchCV) was conducted on the top five models. The GridSearchCV systematically explores all possible combinations of parameters for a given algorithm and evaluates their impact on the model performance. The combination yielding the best outcome is considered optimal (48). In the present study, all algorithms were implemented using the Python package scikit-learn 0.24.1 (37).

### 3.6. Performance Metrics

Accuracy, precision, recall or sensitivity, specificity, F1 score, MCC, Cohen’s kappa statistic (CK or κ), and the area under the receiver operating characteristic curve (AUC-ROC) are all indicators of model performance. The ROC curves, which are based on the sensitivity and specificity of the model, provide valuable graphic representations of the performance of binary classifiers (49). The following Equations 7-13 were utilized to calculate the aforementioned metrics:


Equation 7. $$Accuracy=\frac{TP+TN}{TP+TN+FP+FN}$$

Equation 8. $$Precision=\frac{TP}{TP+FP}$$

Equation 9. $$Sensitivity\;\left(recall\right)=\frac{TP}{TP+FN}$$

Equation 10. $$Specificity=\frac{TN}{TN+FP}$$

Equation 11. $$F1\;score=2\times \frac{Precision\times Recall}{Precision+Recall}$$

Equation 12. $$MCC=\frac{\left(TP\times TN\right)-\left(FP\times FN\right)}{\sqrt{\left(TP+FP\right)\left(TP+FN\right)\left(TN+FP\right)\left(TN+FN\right)}}$$

Equation 13. $$\kappa =\frac{2\times \left(TP\times TN-FP\times FN\right)}{\left(TP+FP\right)\times \left(FP+TN\right)+\left(TP+FN\right)\times \left(FN+TN\right)}$$

Where true positives (TP) stands for true positive, which refers to peptides that have ABF effects and are correctly predicted by the model. True negatives (TN) represents true negative, indicating peptides with no ABF effects that are correctly classified by the model. False positives (FP) denotes false positive results, representing peptides with ABF effects that the model incorrectly classifies as ABF peptides. False negatives (FN) signifies ABF peptides that the model incorrectly classifies as non-ABF peptides. F1-score considers both precision and recall and calculates their harmonic mean. Matthew's correlation coefficient is a metric that remains unaffected by unbalanced datasets. It is the only binary classification rate that yields a high score when the binary classifier accurately predicts the majority of both positive and negative data instances. Also, κ, similar to MCC, considers all four categories of the confusion matrix, including TP, TN, FP, and FN. These two metrics share similarities, with both having a minimum value of -1 for the worst prediction and a maximum value of +1 for the best prediction (50).

## 4. Results and Discussion

### 4.1. Compositional and Positional Analyses

**Figure 3.**
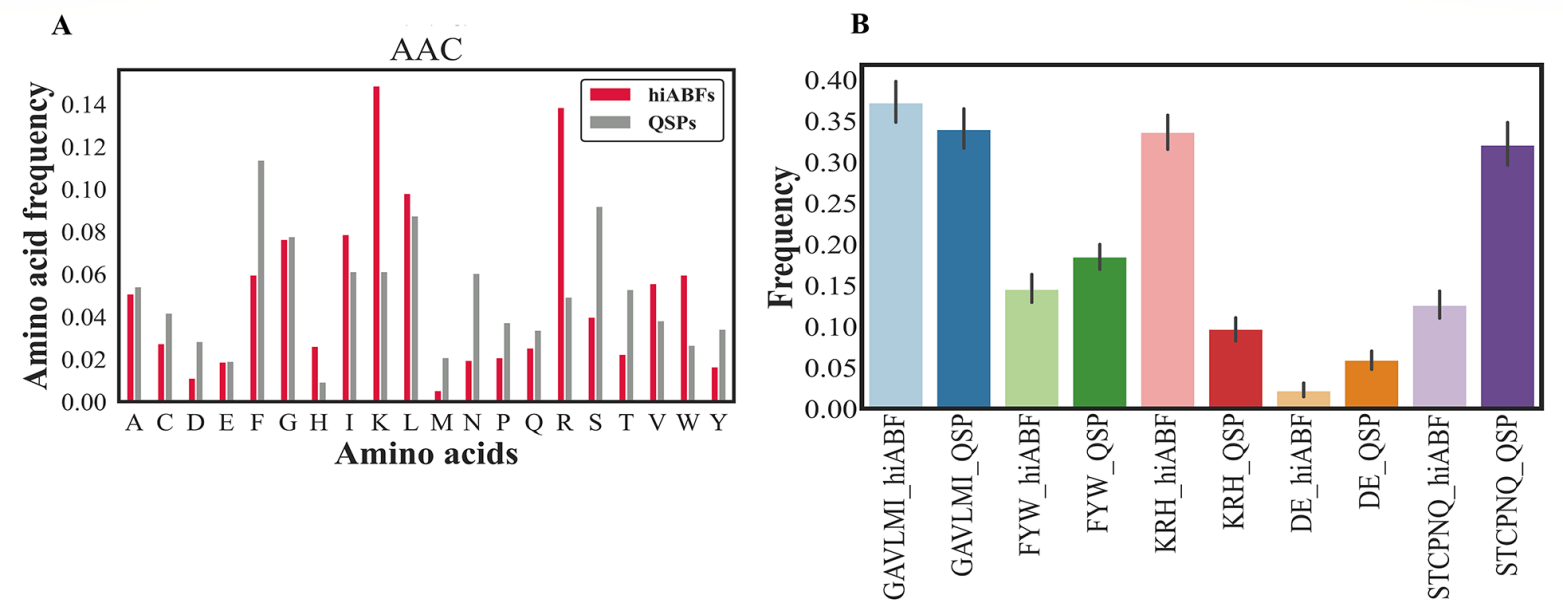
A, comparison of amino acid composition (AAC) between highly active antibiofilms (hiABFs) and quorum-sensing peptides (QSPs); B, the figure demonstrates the distribution of aliphatic, aromatic, positively charged, negatively charged, and uncharged amino acids in hiABFs and QSPs.

A comparison of AAC between hiABFs and QSPs was conducted in this study, the results of which are shown in Figure 3A. It can be observed that hiABFs exhibited a higher abundance of positively charged amino acids, such as arginine (R) and lysine (K), as well as amino acids with hydrophobic side chains, including leucine (L), tryptophan (W), and valine (V), compared to QSPs. Appendix 4 in the supplementary file provides a classification of amino acids, based on their side chain properties. Twenty standard amino acids were categorized into five groups based on their properties. The aliphatic group was composed of G, A, V, L, M, and I amino acids, while the aromatic group consisted of F, Y, and W amino acids. The positive charge group included K, R, and H amino acids, while the negative charge group comprised D and E amino acids. Also, the uncharged group included S, T, C, P, N, and Q amino acids. The distribution of these groups between hiABFs and QSPs is presented in Figure 3B.

**Figure 4.**
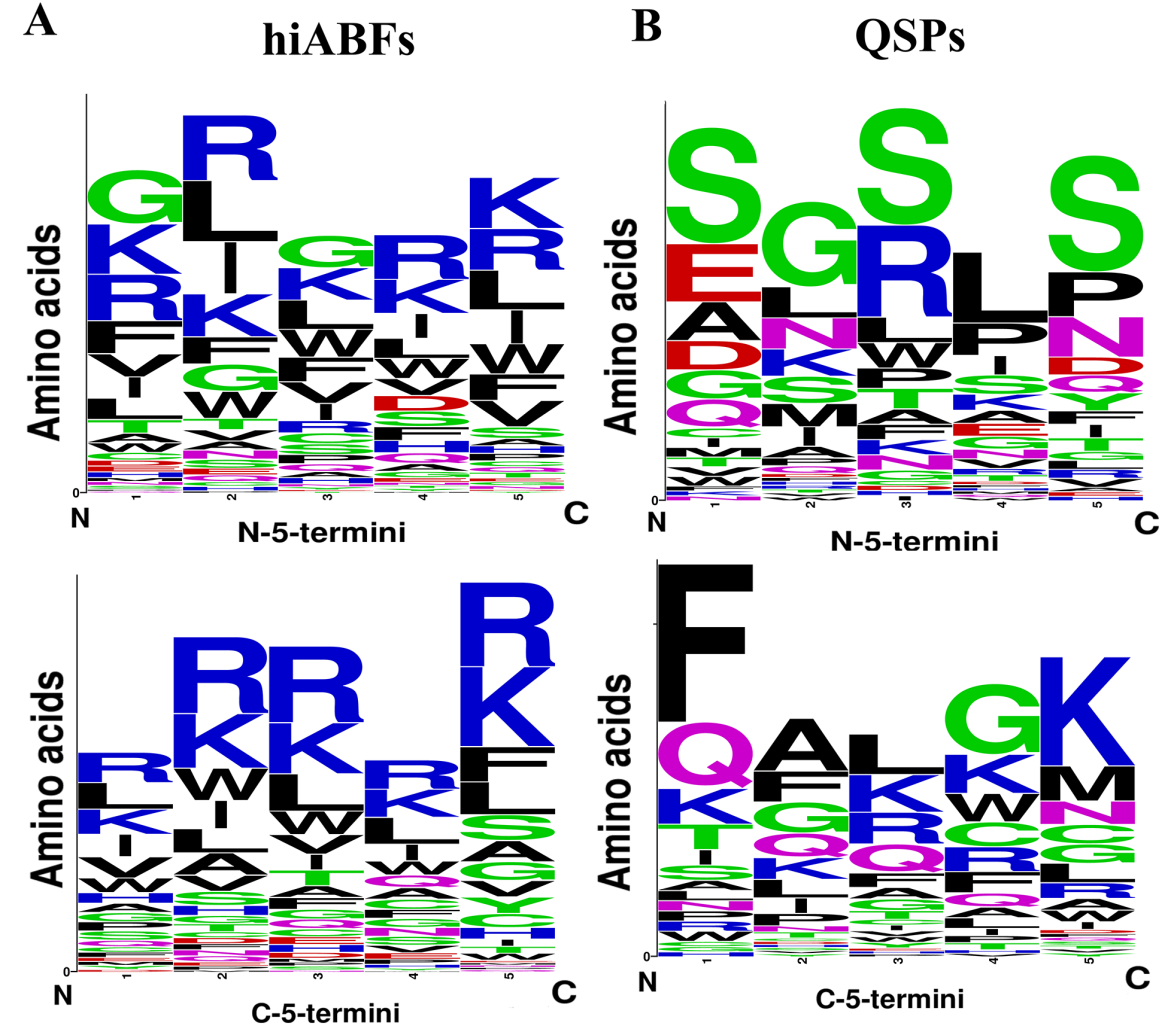
The sequence logos of the first five positions of N and C termini in A, highly active antibiofilms (hiABFs); and B, quorum-sensing peptides (QSPs). The size of residues is proportional to their propensity.

The bacterial membrane contained a significant number of negatively charged components, which made it favorable for interactions with positively charged peptides through cation-pi interactions. In this regard, Segev-Zarko et al. demonstrated that a high frequency of leucine (L) and lysine (K) in peptide sequences could enhance their ABF effects (51). Positional preference analyses were conducted for a comprehensive assessment of peptide sequences, exhibiting high ABF activity. The analysis focused on the first five positions of both N and C termini. Sequences with a length exceeding 10 residues were selected from hiABF and QSP datasets. Figure 4A and B depict the results of positional preference analysis.

In hiABFs, the N-terminal positions were predominantly occupied by arginine (R) and lysine (K), followed by hydrophobic amino acids, including leucine (L) and isoleucine (I). In QSPs, the first position was predominantly occupied by the uncharged and polar amino acid, serine (S), along with negatively charged amino acids, including aspartate (D) and glutamate (E). Serine also exhibited dominance in the third and fifth positions, while glycine (G) and leucine (L) were more frequently observed in the second and fourth positions.

In the C-terminus of hiABFs, arginine (R) and lysine (K) were the most preferred residues in all five positions. In contrast, in QSPs, the first to fourth positions were predominantly occupied by nonpolar amino acids, including phenylalanine (F), alanine (A), leucine (L), and glycine (G). In the fifth position, positively charged residue lysine (K) was predominant. In this regard, a study by Rydberg et al. focused on three peptide sequences, which were exclusively composed of arginine (R) and tryptophan (W). Their findings revealed that an increase in the frequency of arginine in both N-terminus and C-terminus of peptides led to a significant reduction in their cytotoxicity against CHO cells compared to the sequence with a high frequency of tryptophan (W) in the N-terminal position (48).

The ABF peptides have been found to be effective in preventing biofilm formation through the inhibition of the stringent response molecule, ppGpp (11). In a study by Jiale et al., it was discussed that the interaction between the 1018M peptide and ppGpp was influenced by specific amino acids, with valine (V) and arginine (R) playing a crucial role in this interaction (52). Moreover, Shang et al. demonstrated that ABF peptides containing tryptophan residues could disrupt quorum sensing and effectively inhibit biofilm formation in multidrug-resistant *Pseudomonas aeruginosa*. These peptides also exhibited synergistic effects when combined with antibiotics, such as ceftazidime and piperacillin (53).

For further investigation, a comparison was made between hiABFs and peptide sequences with only ABP effects and less than 25% ABF activity, as reported in DRAMP 2.0 and BaAMP databases (54). Appendix 5 presents a comparison of amino acid frequencies. The analysis revealed that the frequency of lysine (K), leucine (L), arginine (R), and tryptophan (W) in hiABFs was significantly higher than ABP peptides, which lacked ABF activity or exhibited no significant activity. This analysis highlighted the significance of lysine (K), leucine (L), arginine (R), and tryptophan (W) residues in interactions with bacterial membranes and other mechanisms involved in biofilm development, such as quorum sensing and ppGpp.

**Figure 5.**
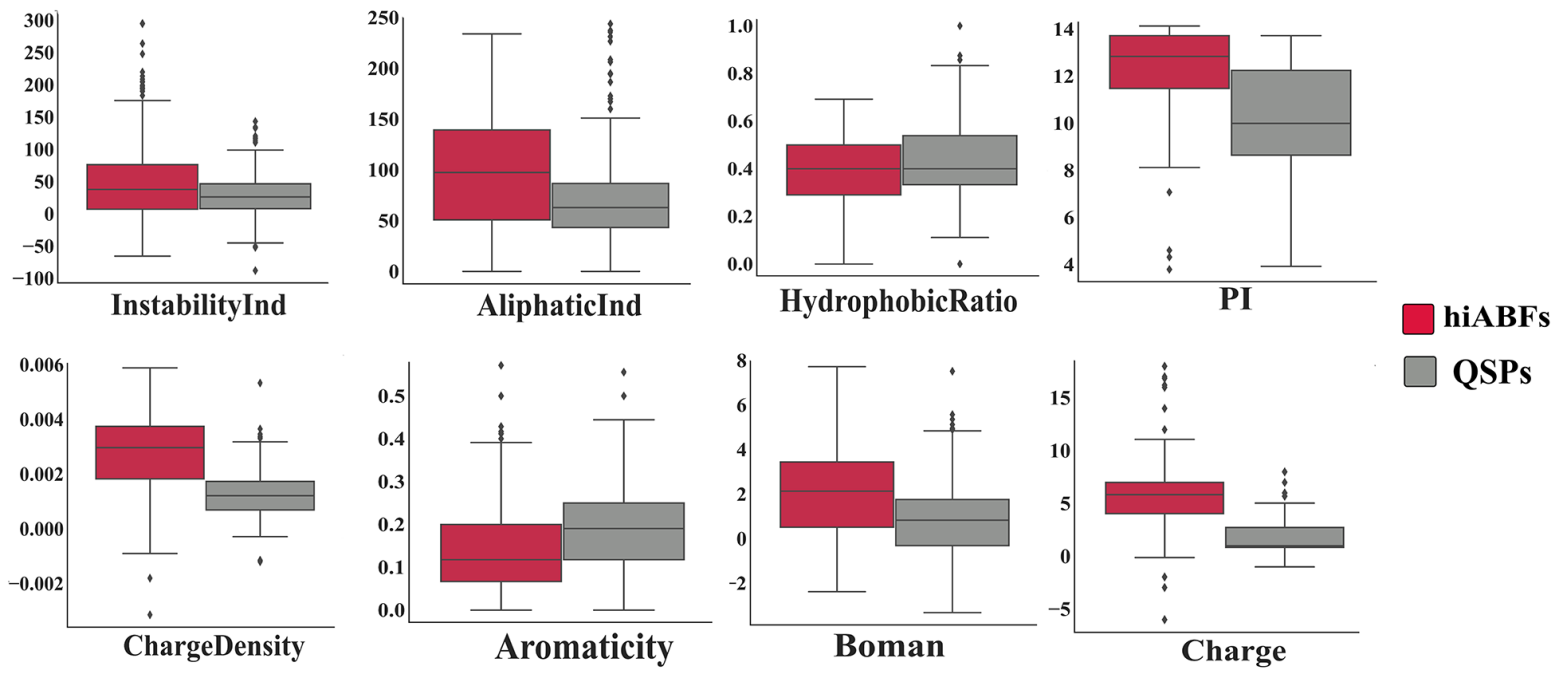
Comparison of physicochemical properties between highly active antibiofilms (hiABFs) and quorum-sensing peptides (QSPs)

An intriguing compositional analysis was conducted to compare peptide sequences that were experimentally validated to have over 50% activity against preformed biofilms (24 hours old) with sequences that influenced biofilm formation when microbial cells were exposed to them for 3 - 24 hours. The categorization of preformed and formation groups was based on the method described by Di Luca et al. (19). Based on the results, it was observed that the formation group exhibited a higher frequency of tryptophan (W), valine (V), and arginine (R), compared to the preformed group. The AAC analysis for both groups is depicted in Appendix 6. The increased prevalence of W, V, and R amino acids in ABF peptides that function during the formation stage is probably attributable to the enhancement of interactions between peptide sequences and ppGpp or quorum-sensing molecules. The physicochemical properties, including charge, charge density, hydrophobic ratio, PI, aliphatic index, aromaticity, Boman index, and instability index of hiABFs and QSPs, were calculated; the statistical results are presented in Figure 5. 

The mean positive charge of hiABFs was found to be higher than that of QSPs. A higher positive charge facilitates electrostatic interactions between peptide sequences and the negatively charged target membrane. As mentioned earlier, optimization strategies employed to enhance the antimicrobial performance of peptides against planktonic cells may not be applicable to peptides with ABF activity. Alginate is one of the major polysaccharides in the biofilm architecture of *P. aeruginosa* and other pulmonary pathogens (1). Stark et al. suggested that increasing the hydrophobicity of cationic peptides could enhance their antibacterial effects (55). However, Benincasa et al. found that when peptide sequences were exposed to an environment containing alginate, an increase in hydrophobicity could lead to peptide aggregation and subsequent deactivation (56). Figure 5 illustrates a comparison of hydrophobicity between hiABFs and QSPs, revealing that hiABFs exhibited lower hydrophobicity compared to QSPs.

Moreover, when comparing hiABFs with ABPs, it was observed that the mean Eisenberg hydrophobicity for hiABFs with an experimentally confirmed high ABF activity was -0.19, while for ABPs, it was 0.11. In an experimental study focusing on IDR-1018 and 1018M peptides against methicillin-resistant *Staphylococcus aureus* (MRSA), it was found that 1018M peptides inhibited biofilm formation, whereas IDR-1018 did not influence biofilm formation. Interestingly, the 1018M peptide exhibited significantly lower hydrophobicity compared to IDR-1018, with its hydrophobic ratio being 25% lower than that of IDR-1018 (52).

Appendix 7 illustrates the comparison results of charge between ABP and ABF peptides. The analysis demonstrated that the average positive charge of ABF sequences was higher than that of antibacterial sequences. This higher positive charge in ABF peptides can be interpreted from another perspective. The biofilm matrix is known to contain extracellular DNA (eDNA) as a prominent component (3). It has been proposed that eDNA plays a critical role in maintaining the structural integrity of biofilms (1). In a study by Mulcahy et al., the chelator-like properties of eDNA were reported (57). Positively charged ABF peptides exhibited a strong affinity for interacting with eDNA within the biofilm matrix. This interaction had the potential to saturate the cation-binding capacity of eDNA and potentially disrupt the eDNA-mediated resistance mechanisms of bacteria in the biofilm state (1).

**Figure 6.**
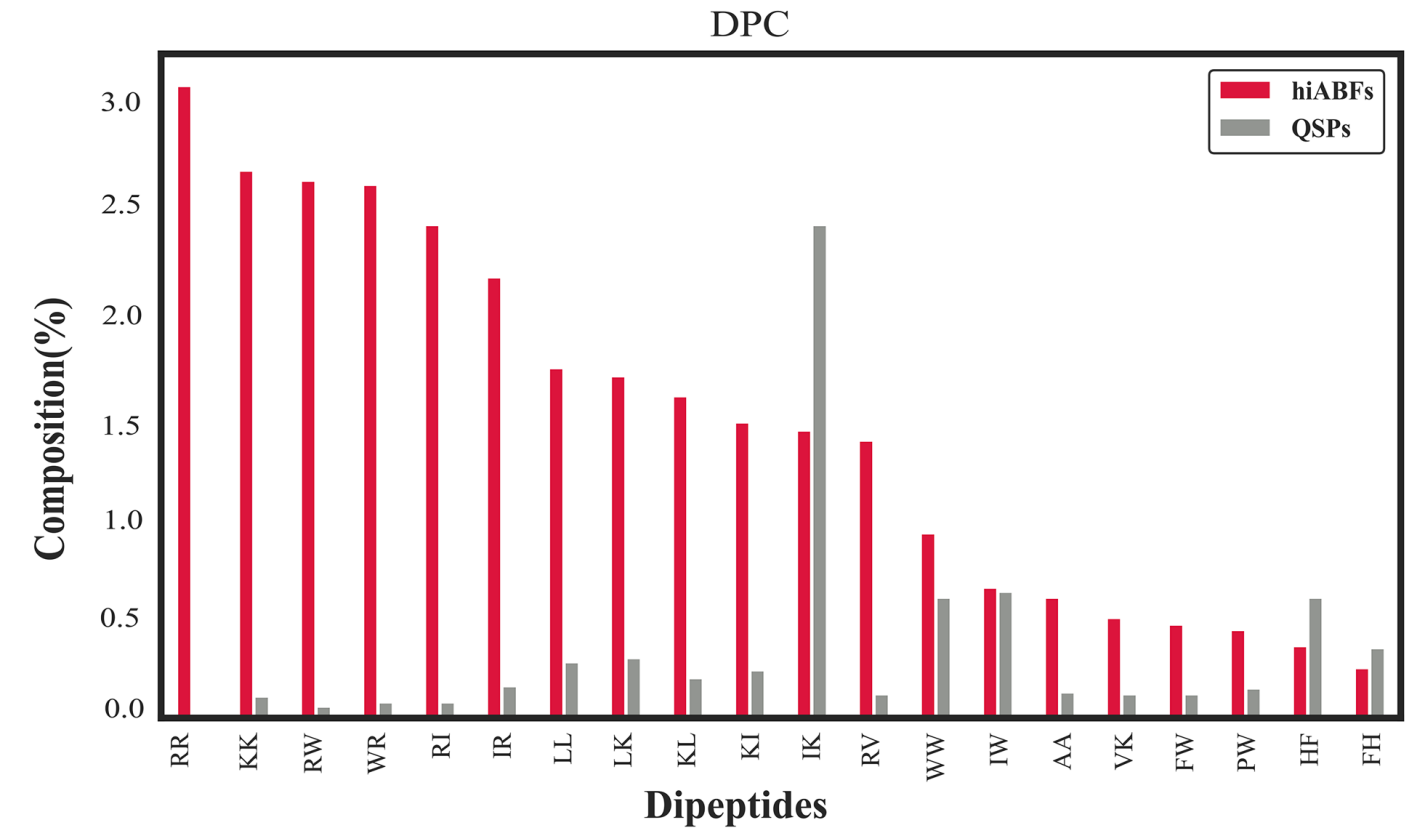
Dipeptide composition (DPC) in comparison between highly active antibiofilms (hiABFs) and quorum-sensing peptides (QSPs)

The dipeptide compositional analysis between hiABFs and QSPs highlighted several prominent dipeptide combinations, including RR, KK, RW, RI, IR, LL, LK, KL, KI, and RV, which were found to be the most abundant DPCs in hiABFs. The analysis of dipeptide compositions revealed the notable occurrence of charged and hydrophobic motifs in ABF peptides with significant ABF effects. This observation aligns with the findings reported by Bose et al., which emphasized that these dipeptide combinations reflected the amphipathic characteristics of ABFs (58). Figure 6 provides a comparative illustration of dipeptide compositions between hiABFs and QSPs, further elucidating the distinguishing patterns and frequencies of di-peptides in these peptide categories.

### 4.2. Feature Selection and Model Performance Evaluation

The performance of all 13 binary classifiers was assessed on both training and validation datasets, using 10-fold cross-validation. This process was repeated 50 times to ensure the robustness of the results. The cross-validation score, which is a reliable metric for evaluating the model performance on unseen data, was utilized for model selection. To gauge variability in performance, the standard deviation of cross-validation scores was computed and considered during the model selection process. Appendix 8 provides a summary of the overall performance of all models. The comparison of models in terms of accuracy is illustrated in Appendix 9.

Among the classifiers, the model based on SVM, random forest, and logistic regression demonstrated superior performance compared to the other classifiers. The logistic regression model achieved an accuracy of 99% on the training set and 93% on the validation set. Also, the random forest model achieved an accuracy of 99% on the training set and 94% on the validation set. Based on the findings, SVM models with different kernels (RBF, poly, and linear) showed comparable performance, with an overall accuracy of 98% on the training set and 93% on the validation set. Considering their high accuracy and consistent performance, classifiers, including logistic regression, random forest, and SVM, were selected for further optimization and analysis.

**Figure 7.**
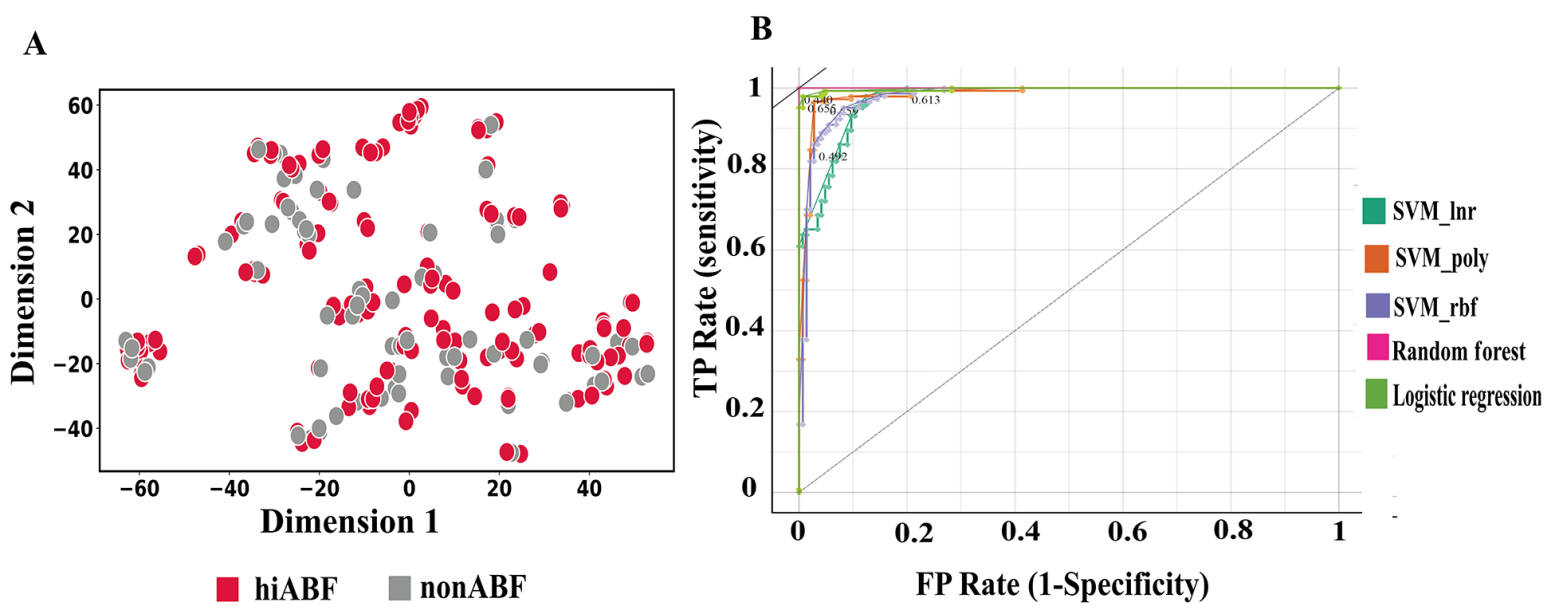
The t-distributed stochastic neighbor embedding (t-SNE) visualization of feature-selected data (A); the ROC curve illustrates the ability of the selected classifier to distinguish between highly active antibiofilms (hiABFs) and quorum-sensing peptides (QSPs).

In the feature space optimization process, both filter-based and wrapper-based methods were employed. First, an MC analysis was conducted with a threshold of 0.75, resulting in the elimination of 230 highly correlated features from the initial feature space. The SelectKbest method was then applied, exploring various K values, ranging from 50 to 200. However, the best results were obtained when K was set at 100. Finally, recursive feature elimination, cross-validated (REFCV) was performed on the selected models. Figure 7A presents the t-SNE visualization of the feature-selected data.

The perplexity parameter was set at 5.0, and the learning rate parameter was set at 200, based on empirical observations and experimentation to obtain significant visualizations. The perplexity parameter in t-SNE plays a crucial role in determining the effective number of neighbors considered for each data point during the dimensionality reduction process (38). In the resulting t-SNE plot, clear separation between data points is not readily apparent. Despite the lack of distinct clusters, the selected classifiers were able to exploit subtle patterns and interact with feature combinations that were not visually discernible in the t-SNE plot. These classifiers successfully learned complex decision boundaries, enabling accurate predictions even in scenarios where the data points were not well-separated within the low-dimensional space. The performance of the optimized models, including the results of hyperparameter tuning and feature selection methods, was evaluated using 10-fold cross-validation on the validation set. The results of analysis, which considered different feature spaces, are presented in Table 2. 

**Table 2.** The Performance Metrics for the Selected Classifiers with Tuned Hyperparameters

Model	Hyperparameters	Method of Feature Selection	Accuracy	Precision	MCC	CK	F1-Score	AUC-ROC
**Logreg**	C': 1000, 'penalty': 'l2', 'solver': 'newton-cg', 'tol': 0.01	MO (75%)	0.98	0.97	0.958	0.958	0.98	0.973
		REFCV	0.99	0.99	0.986	0.986	0.99	0.993
		SelectKbest	0.982	0.98	0.965	0.965	0.98	0.982
**RFC**	'min_samples_leaf': 2, 'n_estimators': 500, 'max_depth': 8, 'max_features': 'sqrt'	MO (75%)	0.982	0.98	0.965	0.965	0.98	0.982
		SelectKbest	0.97	0.97	0.947	0.944	0.97	0.972
		REFCV	0.97	0.97	0.9375	0.9375	0.97	0.9688
**SVM_rbf**	'C': 100, 'gamma': 0.001	MO (75%)	0.985	0.99	0.971	0.979	0.99	0.98
		SelectKbest	0.97	0.975	0.944	0.942	0.97	0.973
**SVM_poly**	'C': 0.1, 'degree': 2, 'gamma': 1	MO (75%)	0.98	0.96	0.958	0.958	0.98	0.978
		SelectKbest	0.98	0.98	0.953	0.958	0.98	0.948
**SVM_lnr**	'C': 0.1, 'gamma': 1	MO (75%)	0.98	0.99	0.972	0.972	0.99	0.986
		REFCV	0.99	0.99	0.976	0.979	0.99	0.99
		SelectKbest	0.96	0.96	0.916	0.916	0.96	0.957

Abbreviations: Logreg, logistic regression; RFC, random forest classifier; SVM, support vector machine; MCC, Matthew's correlation coefficient; CK, Cohen’s kappa statistic; AUC-ROC, area under the receiver operating characteristic curve; REFCV, recursive feature elimination, cross-validated.

In Figure 7B, the ROC curves for models with superior performance are displayed. These curves were plotted using the Orange 3.33.0 Python package (59).

Diverse metrics were used for the analysis and comparison of model performance. The numbers of positive and negative libraries were 183 and 198, respectively. Consequently, the dataset sizes were comparable and almost balanced, enabling the use of accuracy as a criterion for assessing the model performance. To mitigate the potential for overly optimistic reporting of model performance, the MCC value was also taken into consideration. Equation 12 demonstrates that MCC yields a high score only when the binary classifiers perform well in all categories of the confusion matrix, including TP, FN, TN, and FP, proportionally adjusted to the size of positive and negative libraries (41). Table 2 presents the results of analysis based on the MCC value, which demonstrate the strong performance of classifiers in predicting the high ABF activity of peptide sequences.

### 4.3. Model Performance on the Independent Test Set

Table 3 displays the performance of the optimized classifiers on the independent test set.

**Table 3.** The Performance of Optimized Models on the Independent Test Set

Model	Accuracy	Precision	MCC	CK	F1-score	AUC-ROC
**Logreg**	0.989	0.976	0.979	0.979	0.988	0.999
**RF**	0.989	0.99	0.979	0.972	0.99	0.988
**SVM**						
RBF	0.99	0.99	0.979	0.979	0.98	0.99
Polynomial	0.96	0.96	0.917	0.917	0.96	0.958
Linear	0.98	0.99	0.876	0.876	0.942	0.936

Abbreviations: Logreg, logistic regression; RF, random forest; SVM, support vector machine; MCC, Matthew's correlation coefficient; CK, Cohen’s kappa statistic; AUC-ROC, area under the receiver operating characteristic curve.

The high ABF activity of peptide sequences can be predicted with high accuracy and precision using the optimized models, based on logistic regression, random forest, and support vector machine (RBF kernel) trained on the proposed feature space. The logistic regression-based model achieved an accuracy of 98.9% and a precision of 97.6%, while the random forest-based model achieved an accuracy of 98.9% and a precision of 99%. The SVM-based model, with an RBF kernel, achieved the highest accuracy (99%) and precision (99%). Compared to similar models, BIOFIN utilized SVM and random forest-based classifiers on different amino acid compositions and reported an accuracy of 97% and an MCC of 0.83 (20). The frequency of amino acids was employed by dPABBs in combination with SVM and Weka algorithms, resulting in accuracy and MCC values of 95.2% and 0.91, respectively (19). Fallah Atanaki et al. developed an SVM-based model that achieved an accuracy of 95% and an MCC of 0.89 (16). The model was trained separately with each feature set. All feature sets exhibited good potential for distinguishing between hiABFs and QSPs, except for AAC. However, the composition of all groups yielded better results.

### 4.4. Conclusions

In recent decades, the characteristics of infections have undergone fundamental changes, primarily due to antibiotic resistance caused by bacterial biofilms. Consequently, a significant emphasis has been placed on developing antimicrobial agents that can address the challenges posed by antibiotic resistance. Peptides have emerged as a promising class of antimicrobial biomolecules, with ABF peptides showing remarkable potential in eradicating preformed biofilms or inhibiting biofilm formation. Meanwhile, experimental methods for designing and synthesizing ABF peptides can be cumbersome. Therefore, development of computational methods to streamline this process has become indispensable. The application of machine learning and artificial intelligence in drug discovery and development has gained significant attention due to its advantages. Consequently, there has been a rapid increase in the number of studies utilizing these techniques for peptide prediction and design. In this study, the advantages of multiple machine learning algorithms were utilized to create a computational platform for predicting the high ABF effect of peptide sequences. The focus was on ABF peptides with significant activity due to a lack of research incorporating significant ABF activity in their datasets and model development. The dataset gathering process involved the inclusion of peptide sequences with ABF activity equal to or higher than 50%. As for the negative datasets, QSPs were utilized. Duplicated sequences, sequences containing non-standard amino acid residues, and sequences with any modifications or indefinite residues were excluded from the datasets. The feature space was created by calculating features based on physicochemical properties, amino acid composition, order, and their distribution. Filter- and wrapper-based feature selection methods were employed to construct the feature space. A range of binary classifiers with 10-fold cross-validation was used to identify models with superior performance, which were subsequently optimized by adjusting their hyperparameters using GridSearchCV. Among the selected models, those based on logistic regression, SVM, and random forest demonstrated better performance on both training and validation datasets in terms of accuracy, precision, and MCC. The model performance and the created feature space were evaluated on an independent test set to predict highly active ABF properties. The model achieved 99% accuracy, 99% precision, and an MCC of 0.979. Overall, an in-depth analysis of the structure, composition, amino acid preferences, and relationship with the mechanism of action in hiABFs can greatly facilitate the design of peptide-based therapeutics. While computational methods play a crucial role in streamlining the drug design and development process, it is important to acknowledge that there is a long way ahead before computer algorithms can lead to the development of FDA-approved drugs.

ijpr-22-1-138704-s001.pdf

## Data Availability

The data and code scripts presented in this study will be openly available at hiABF after publication.
