# Supplementary material for: Integration of Machine Learning and Structural Analysis for Predicting Peptide Antibiofilm Effects: Advancements in Drug Discovery for Biofilm-Related Infections
Source: Iran J Pharm Res. 2023 Sep 30;22(1):e138704. doi: 10.5812/ijpr-138704 (PMC10916117; doi:10.5812/ijpr-138704)
Supplement: ijpr-22-1-138704-s001.pdf [file ijpr-22-1-138704-s001.pdf]

Supplementary Information

Amino acid groups and distributions based on charge, hydrophobicity, normalized van der Waals volume, polarity, polarizability, secondary structure, and solvent accessibility are presented in Appendix 1.

Appendix 1. Division of amino acids in to three groups based on their physicochemical properties

**Property**	**Class 1**	**Class 2**	**Class 3**
**Charge**	**Neutral**	**Negatively charged**	**Positively charged**
Residues	*A, C, F, G, H, I, L, M, N, P, Q, S, T, V, W, Y*	*D, E*	*K, R*
**Hydrophobicity**	**Hydrophobic**	**Neutral**	**Polar**
Residues	*C, F, I, L, M, V, W*	*A, G, H, P, S, T, Y*	*D, E, K, N, Q, R*
**Normalized van der Waals volume**	**Volume range: 0-2.78**	**Volume range: 2.95-4.0**	**Volume range: 4.03-8.08**
Residues	*A, C, D, G, P, S, T*	*E, I, L, N, Q, V*	*F, H, K, M, R, W, Y*
**Polarity**	**Polarity range: 4.9-6.2**	**Polarity range: 8.0-9.2**	**Polarity range: 10.4-13.0**
Residues	*C, F, I, L, M, V, W, Y*	*A, G, P, S, T*	*D, E, H, K, N, Q, R*
**Polarizability**	**Polarizability range: 0 - .108**	**Polarizability range 0.128-0.186**	**Polarizability range 0.219-0.409**
Residues	*A, D, G, S, T*	*C, E, I, L, N, P, Q, V*	*F, H, K, M, R, W, Y*
**Secondary Structure**	**Coil**	**Helix**	**Strand**
Residues	*D, G, N, P, S*	*A, E, H, K, L, M, Q, R*	*C, F, I, T, V, W, Y*
**Solvent Accessibility**	**Buried**	**Intermediate**	**Exposed**
Residues	*A, C, F, G, I, L, V, W*	*H, M, P, S, T, Y*	*D, E, K, N, R, Q*

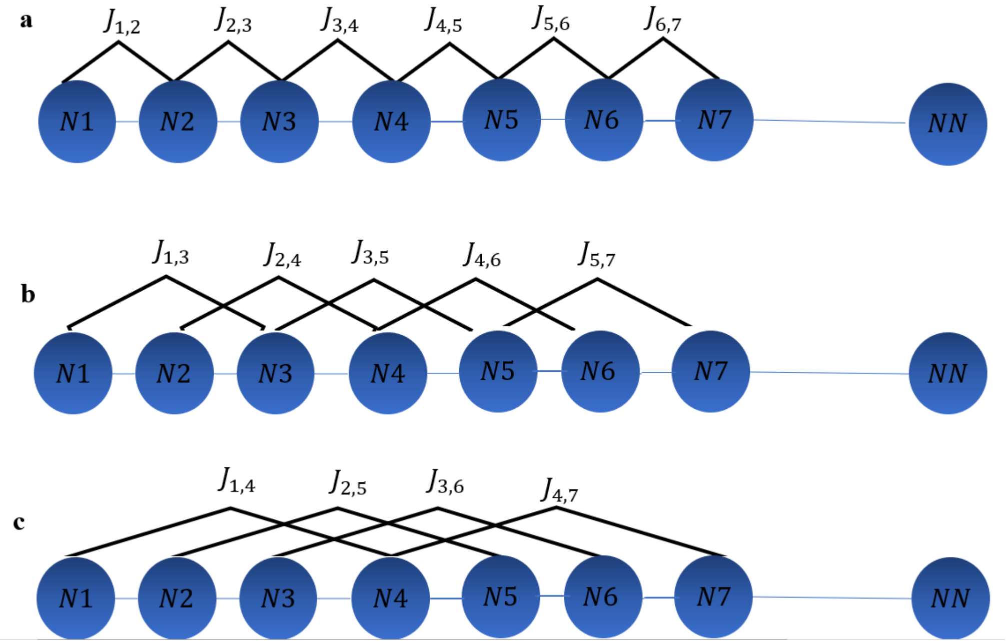

Appendix 2. Schematic presentation of the a) first-ranked, all the most contiguous residues, b) second-ranked, the second most contiguous residues, and c) third-ranked, the third most contiguous residues sequence-order-coupling model in peptide sequences with length equal to N.

A list of global and peptide descriptors and their definitions are summarized in Appendix 3.

Appendix 3. Summary of Global and pepdescriptors

Features	Description
**Aromaticity**	This feature is calculated based on the relative frequency of amino acids with aromatic R groups; Phenylalanine+Tyrosine+Tryptophan (1).
**Aliphatic Index**	Aliphatic index is based on the correlation between thermal stability and the aliphatic index of a protein. This descriptor is calculated based on the frequency of the alanine, Valine, Leucine and Isoleucine. Aliphatic index = X(Ala)+a X(Val)+b X(Ile)+X(Leu), where X represents the molar percentage of these amino acids, a and b is the side chain volume of the Valine, Leucine and Isoleucine. For Val a = 2.9 and for Leu and Ile b = 3.9 (2).
**Boman Index**	This index is based on protein-protein interaction. Indeed, high Boman index indicates that the peptide has a high potential for interaction with other proteins (3).
**Hydrophobic Ratio**	This feature is calculated based on the relative frequency of Ala, Cys, Phe, Ile, Leu, Met, Val (4).
**Hydrophobicity**	This descriptor is calculated based on Argos, Hopp-woods, Janin, Gravy, Kyte-Doolittle, and Eisenberg hydrophobicity scale (5–9).
**Instability Index**	By studying several stable and unstable proteins, Guruprasad *et al*. found that some dipeptides in unstable proteins are significantly different from stable proteins. Based on their effects on protein instability, an instability index is assigned for each. Summation of these indexes normalized on the protein sequences and used as a benchmark for a protein or peptide instability (10).
**Length**	Peptide sequences length
**Molecular weight**	MW of peptide sequences
**Charge**	NetCharge, Calculates charge of peptides sequence at pH 7.4, charge_pysc and charge_acid calculates amino acid charge at pH 7 and acidic pH respectively (11).

**Charge density**	Calculated by dividing charge by molecular weight
**Isoelectric point**	A pH of a solution that a net charge of a peptide sequence is zero
**Bulkiness**	This feature is calculated based on amino acid side chain bulkiness (12)
**Insertion**	E(z) is a residue-based potential for calculating the amino acid side chain insertion propensity into the lipid bilayer (13)
**α-helix propensity**	This descriptor is obtained based on Levitt amino acid alpha helix propensity. Statistical studies of proteins of the known structure have shown that amino acids have clear conformational preferences for one type of secondary structure (14).
**Flexibility**	Amino acid side chain flexibility scale (11).
**Side chain composition, polarity, molecular volume**	Mainly two methods are used to calculate these features: The Grantham method that analyzed amino acid formulation to explain protein evolution, and a PCA-derived scale based on amino acid side chain that uses six different probes from GRID program (15,16).
**Isotropic surface area (ISA) Electronic charge index (ECI)**	ISA and ECI are used as descriptor for amino acids side chains. ISA is the molecule's surface accessible to nonspecific interactions with the solvent; the molecule's surface involved in specific hydrogen bonding with water is not considered. ECI is a measure of molecular polarity (17).
**Polarity**	This feature is based on amino acid polarity scale (11).
**Refractivity**	The refractive index of each amino acid was calculated by McMeekin *et al*. For each protein or peptide, their refractivity is estimated based on the refractive index of each amino acid (11,18).
**Selectivity**	In modlamp, this feature is calculated based on Juretic *et al*.'s method. Their study has been done on antimicrobial peptides' data and assessing their selectivity (19).
**Topology**	The topological shape and size of the amino acid side chain are calculated based on graph theory and weighted connected graph model of amino acid side chain (20).
**Side chain conformation and rotamer**	This descriptor is derived from principal component analysis (PCA) applied to MS-WHIM 3D-description matrices. MS-WHIM indexes are a collection of 36 statistical indexes to extract a molecule's steric and electrostatic 3D properties. Zaliani *et al*. extended MS-WHIM to natural amino acid's side-chain conformation and the rotamer library. Modlamp calculates this feature with MSW function(11,21).
**Transmembrane propensity**	TM propensity is based on hydrophobicity analysis of transmembrane proteins of known structure (22).
**Z-Scale**	These features are based on PCA on 26 peptide descriptors, including NMR, TLC, formation heat, absolute electronegativity, etc. Principal properties were calculated and mainly reflected lipophilicity, steric and electrostatic properties. In modlamp, these descriptors are calculated by Z-Scale function (23).
**Pharmacophoric features**	PepArc is pharmacophoric feature based on hydrophobicity, polarity, positive charge, negative charge and proline. PPCALI is a descriptor obtained by PCA on 143 amino acids physicochemical properties and pepCAT is based on pharmacophoric features of side chains (24).

Appendix 4. Amino acids' R-group properties

**Amino acids**	**Properties**
**Glycine (Gly, G)**	Nonpolar aliphatic R-group
**Alanine (Ala, A)**	
**Proline (Pro, P)**	
**Valine (Val, V)**	

**Leucine (Leu, L)**	
**Isoleucine (Ile, I)**	
**Methionine (Met, M)**	
**Lysine (Lys, K)**	Positively charged R-group
**Arginine (Arg, R)**	
**Histidine (His, H)**	
**Phenylalanine (Phe, F)**	Aromatic R-group
**Tyrosine (Tyr, Y)**	
**Tryptophan (Trp, W)**	
**Aspartate (Asp, D)**	Negatively charged R-group
**Glutamate (Glu, E)**	
**Serine (Ser, S)**	Polar, uncharged R-group
**Threonine (Thr, T)**	
**Cysteine (Cys, C)**	
**Asparagine (Asn, N)**	
**Glutamine (Gln, Q)**	

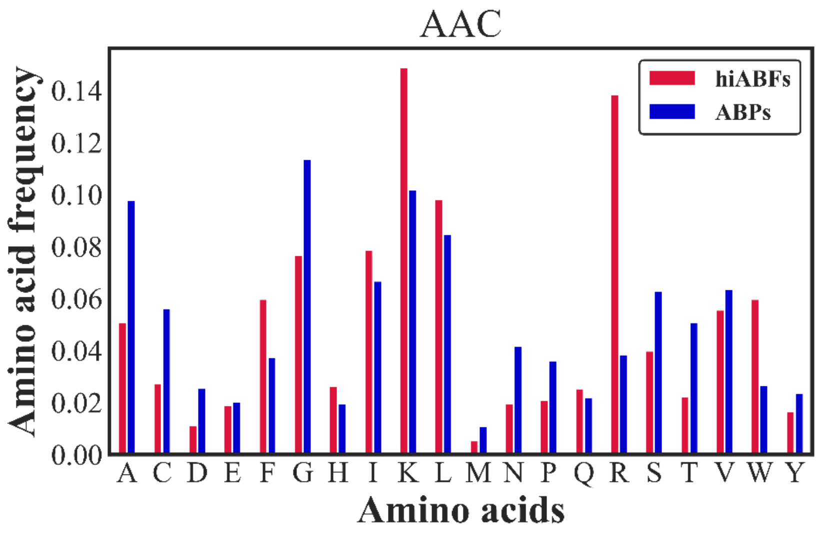

Appendix 5. Comparison between amino acid frequencies between hiABFs and ABPs

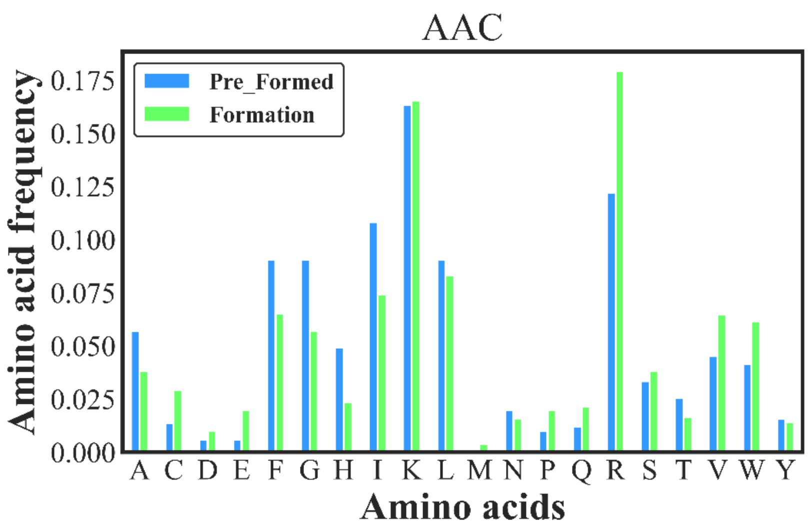

Appendix 6. Comparison between amino acid frequencies between preformed and Formation group.

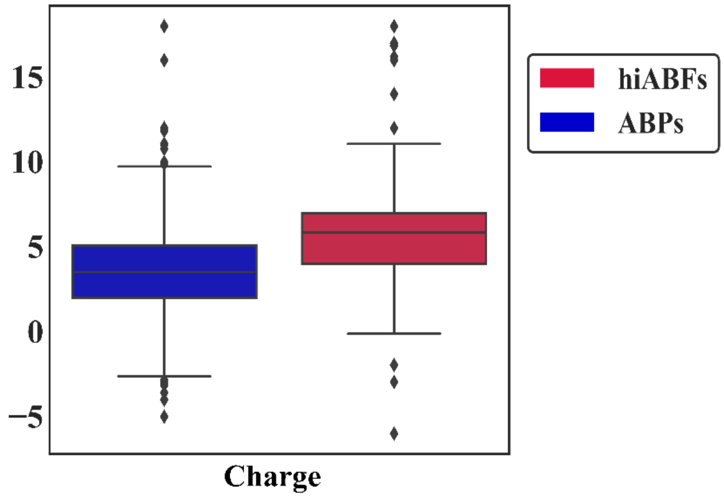

Appendix 7. Comparison of charge between Antibacterial Peptides (ABPs) and Antibiofilm Peptides (ABFs).

The performance metrics of binary classifiers on training and validation data with ten-fold cross-validation are presented in Appendix 8 and Appendix 7.

Appendix 8. Binary classifiers' performance metrics, Based on 10-fold cross validation

Classifier	CV (std)	A	P	R	MCC	CK	ROC-AUC score	F1-score
**LOGREG**	2.29%	99%	99%	99%	97.18%	97.18%	98.59%	99%
		93%	93%	92%	82.92%	82.61%	90.71%	93%
**CART**	5.85%	92%	90%	90%	84.31%	83.96%	92.46%	90%
		83.5%	83%	80%	65.47%	65.22%	82.14%	80%
**RF**	2.07%	99%	99%	99%	98.60%	98.59%	99.21%	99%
		94%	94%	94%	87.13%	87.05%	93.21%	93%
**GBC**	2.78%	97%	97%	97%	96.47%	96.47%	98.19%	98%
		85%	83%	81%	69.95%	69.34%	83.93%	82%
**ADC**	3.10%	97%	96%	96%	95.92%	95.92%	96.9%	96%
		91%	92%	91%	80.23%	80.35%	91.43%	92%
**LDA**	8.31%	75%	70%	71%	51.01%	50.65%	75.71%	74%
		75%	74%	60%	48.08%	46.27%	72.14%	70%
**QDA**	9.23%	61%	60%	61%	22.53%	22.31%	61.24%	60%
		55%	60%	55%	4.93%	4.83%	52.5%	50%
**NB**	11.93%	72%	70%	75%	44.60%	43.64%	72.40%	71%
	17.78%	67%	62%	62%	37.14%	35.14%	68.57%	67%
**SVC_lr**	2.29%	99%*	98%	98%	97.18%	97.18%	98.59%	98%
		93%	93%	93%	82.86%	82.86%	91.43%	93%
**SVC_rbf**	3.18%	98%*	99%	98%	96.48%	96.48%	98.28%	98%
		93%	94%	95%	88.79%	88.10%	93.21%	92%
**SVC_poly**	2.29%	99%*	99%	99%	97.18%	97.18%	98.59%	99%
		92%	92%	94%	87.79%	87.60%	92.21%	92%
**SVC_sig**	3.17%	97%	97%	97%	95.77%	97.88%	97.88%	96%
		85%	82%	82%	70.66%	68.89%	83.21%	80%
**KNN**	5.37%	90%	90%	90%	83.05%	82.98%	91.27%	91%
		91%	90%	90%	83.67%	82.35%	90%	91%

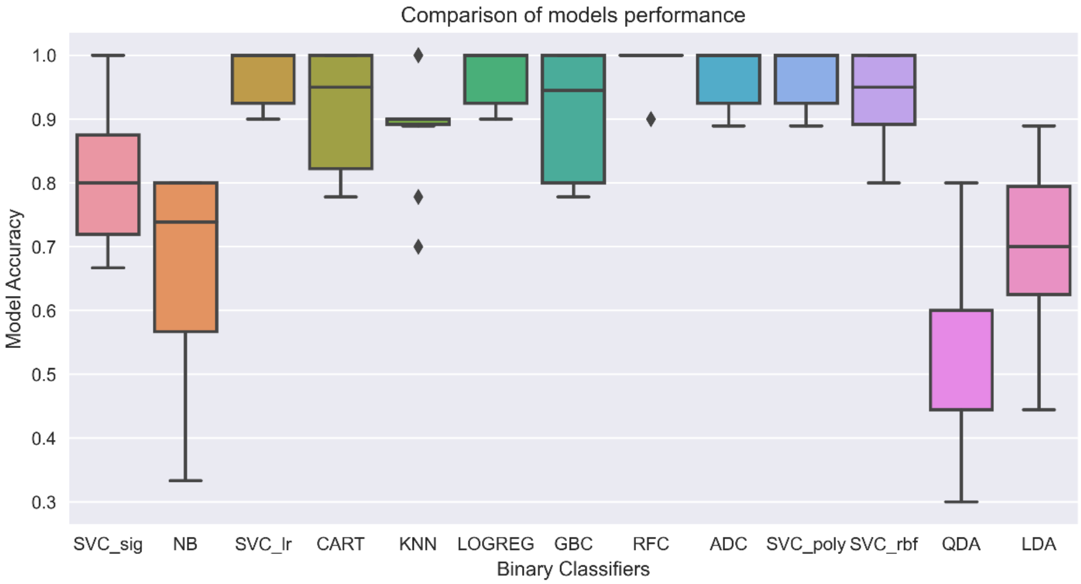

Appendix 9. Comparison of classifiers' performance based on Accuracy

References:

1. Lobry JR, Gautier C. Hydrophobicity, expressivity and aromaticity are the major trends of amino-acid usage in 999 Escherichia coli chromosome-encoded genes. Nucleic Acids Res. 1994 Aug 11;22(15):3174–80.

2. Thermostability and Aliphatic Index of Globular Proteins. The Journal of Biochemistry [Internet]. 1980 Oct [cited 2022 Jul 9]; Available from: https://academic.oup.com/jb/article/88/6/1895/773432/Thermostability-and-Aliphatic-Index-of-Globular

3. Azad MA, Huttunen-Hennelly HEK, Ross Friedman C. Bioactivity and the First Transmission Electron Microscopy Immunogold Studies of Short De Novo-Designed Antimicrobial Peptides ▿ . Antimicrob Agents Chemother. 2011 May;55(5):2137–45.

4. Yin LM, Edwards MA, Li J, Yip CM, Deber CM. Roles of Hydrophobicity and Charge Distribution of Cationic Antimicrobial Peptides in Peptide-Membrane Interactions. J Biol Chem. 2012 Mar 2;287(10):7738–45.

5. Argos P, Rao JKM, Hargrave PA. Structural Prediction of Membrane-Bound Proteins. European Journal of Biochemistry. 1982;128(2–3):565–75.

6. Hopp TP, Woods KR. Prediction of protein antigenic determinants from amino acid sequences. PNAS. 1981 Jun 1;78(6):3824–8.

7. Cornette JL, Cease KB, Margalit H, Spouge JL, Berzofsky JA, DeLisi C. Hydrophobicity scales and computational techniques for detecting amphipathic structures in proteins. Journal of Molecular Biology. 1987 Jun;195(3):659–85.

8. Kyte J, Doolittle RF. A simple method for displaying the hydropathic character of a protein. Journal of Molecular Biology. 1982 May;157(1):105–32.

9. Eisenberg D, Weiss RM, Terwilliger TC, Wilcox W. Hydrophobic moments and protein structure. Faraday Symp Chem Soc. 1982;17:109.

10. Guruprasad K, Reddy BVB, Pandit MW. Correlation between stability of a protein and its dipeptide composition: a novel approach for predicting *in vivo* stability of a protein from its primary sequence. Protein Eng Des Sel. 1990;4(2):155–61.

11. Müller AT, Gabernet G, Hiss JA, Schneider G. modlAMP: Python for antimicrobial peptides. Bioinformatics. 2017 Sep 1;33(17):2753–5.

12. Zimmerman JM, Eliezer N, Simha R. The characterization of amino acid sequences in proteins by statistical methods. Journal of Theoretical Biology. 1968 Nov;21(2):170–201.

13. Senes A, Chadi DC, Law PB, Walters RFS, Nanda V, DeGrado WF. Ez, a Depth-dependent Potential for Assessing the Energies of Insertion of Amino Acid Side-chains into Membranes: Derivation and Applications to Determining the Orientation of Transmembrane and Interfacial Helices. Journal of Molecular Biology. 2007 Feb;366(2):436–48.

14. Koehl P, Levitt M. Structure-based conformational preferences of amino acids. PNAS. 1999 Oct 26;96(22):12524–9.

15. Grantham R. Amino Acid Difference Formula to Help Explain Protein Evolution. Science. 1974 Sep 6;185(4154):862–4.

16. Cecchetti V, Filipponi E, Fravolini A, Tabarrini O, Bonelli D, Clementi M, et al. Chemometric Methodologies in a Quantitative Structure−Activity Relationship Study: The Antibacterial Activity of 6-Aminoquinolones. J Med Chem. 1997 May 1;40(11):1698–706.

17. Collantes ER, Dunn WJ. Amino Acid Side Chain Descriptors for Quantitative Structure-Activity Relationship Studies of Peptide Analogs. J Med Chem. 1995 Jul;38(14):2705–13.

18. Zhao H, Brown PH, Schuck P. On the Distribution of Protein Refractive Index Increments. Biophysical Journal. 2011 May;100(9):2309–17.

19. Juretić D, Vukičević D, Ilić N, Antcheva N, Tossi A. Computational Design of Highly Selective Antimicrobial Peptides. J Chem Inf Model. 2009 Dec 28;49(12):2873–82.

20. Raychaudhury C, Banerjee A, Bag P, Roy S. Topological Shape and Size of Peptides: Identification of Potential Allele Specific Helper T Cell Antigenic Sites. J Chem Inf Comput Sci. 1999 Mar 22;39(2):248–54.

21. Zaliani A, Gancia E. MS-WHIM Scores for Amino Acids: A New 3D-Description for Peptide QSAR and QSPR Studies. J Chem Inf Comput Sci. 1999 May 25;39(3):525–33.

22. Zhao G, London E. An amino acid “transmembrane tendency” scale that approaches the theoretical limit to accuracy for prediction of transmembrane helices: Relationship to biological hydrophobicity. Protein Sci. 2006 Aug;15(8):1987–2001.

23. Sandberg M, Eriksson L, Jonsson J, Sjöström M, Wold S. New Chemical Descriptors Relevant for the Design of Biologically Active Peptides. A Multivariate Characterization of 87 Amino Acids. J Med Chem. 1998 Jul 1;41(14):2481–91.

24. Koch CP, Perna AM, Pillong M, Todoroff NK, Wrede P, Folkers G, et al. Scrutinizing MHC-I Binding Peptides and Their Limits of Variation. PLoS Comput Biol. 2013 Jun 6;9(6):e1003088.
